# A Comprehensive Performance Analysis of Transfer Learning Optimization in Visual Field Defect Classification

**DOI:** 10.3390/diagnostics12051258

**Published:** 2022-05-18

**Authors:** Masyitah Abu, Nik Adilah Hanin Zahri, Amiza Amir, Muhammad Izham Ismail, Azhany Yaakub, Said Amirul Anwar, Muhammad Imran Ahmad

**Affiliations:** 1 Center of Excellence for Advanced Computing, Faculty of Electronic Engineering Technology, Universiti Malaysia Perlis, Kangar 01000, Malaysia; masyitah@studentmail.unimap.edu.my (M.A.); amizaamir@unimap.edu.my (A.A.); 2 Institute of Engineering Mathematics, Faculty of Applied and Human Sciences, Universiti Malaysia Perlis, Arau 02600, Malaysia; izham@unimap.edu.my; 3 Department of Ophthalmology & Visual Science, School of Medical Sciences, Universiti Sains Malaysia, Kubang Kerian 16150, Malaysia; azhany@usm.my; 4 Faculty of Electronic Engineering Technology, Universiti Malaysia Perlis, Arau 02600, Malaysia; said@unimap.edu.my (S.A.A.); m.imran@unimap.edu.my (M.I.A.)

**Keywords:** VF defect, CNN, hyperparameter, fine-tuning

## Abstract

Numerous research have demonstrated that Convolutional Neural Network (CNN) models are capable of classifying visual field (VF) defects with great accuracy. In this study, we evaluated the performance of different pre-trained models (VGG-Net, MobileNet, ResNet, and DenseNet) in classifying VF defects and produced a comprehensive comparative analysis to compare the performance of different CNN models before and after hyperparameter tuning and fine-tuning. Using 32 batch sizes, 50 epochs, and ADAM as the optimizer to optimize weight, bias, and learning rate, VGG-16 obtained the highest accuracy of 97.63 percent, according to experimental findings. Subsequently, Bayesian optimization was utilized to execute automated hyperparameter tuning and automated fine-tuning layers of the pre-trained models to determine the optimal hyperparameter and fine-tuning layer for classifying many VF defect with the highest accuracy. We found that the combination of different hyperparameters and fine-tuning of the pre-trained models significantly impact the performance of deep learning models for this classification task. In addition, we also discovered that the automated selection of optimal hyperparameters and fine-tuning by Bayesian has significantly enhanced the performance of the pre-trained models. The results observed the best performance for the DenseNet-121 model with a validation accuracy of 98.46% and a test accuracy of 99.57% for the tested datasets.

## 1. Introduction

**Figure 1:**
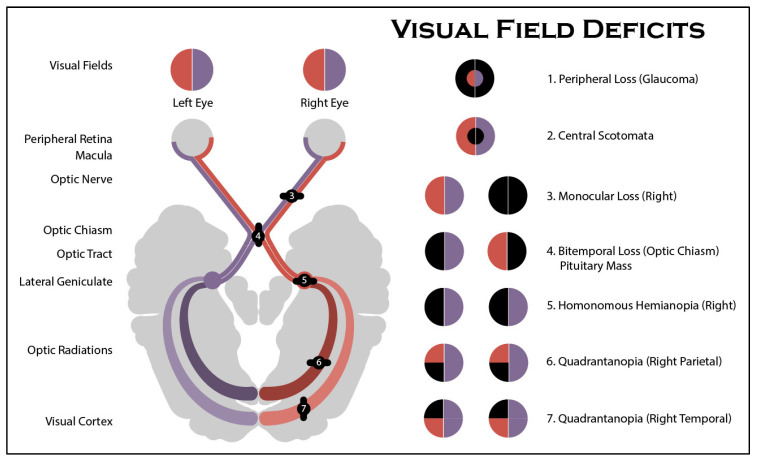
VF Defect Regions [1].

The optic pathway is an anatomical pathway connected to the brain, which sends a signal to the retina of the human vision. The damage to the optic pathway that can result in varied losses in sections of the human vision is known as Visual Field (VF) defects. VF deficits can indicate several serious optic pathway illnesses such as tumors or strokes. The border and extent of defects in the visual field suggest different types of defects and the risk of various optic pathway illnesses. A variety of defects can develop in human vision; for example, Figure 1 shows that lesions can occur in any part of VF. The defect pattern can suggest a variety of disorders. The dark color represents the localization of defects in the visual field. VF defects studied in this work are categorized as follows:

- Right-to-left homonymous hemianopia—A VF defect condition that affects half of the eye, possibly both eyes or only the right and left eyes. Hemianopia can indicate a brain bleed, hemorrhage, tumor, or plus collection.
- Left/right/lower/upper quadrantanopia—A VF defect in the quarter section of the eye at several locations (right, left, higher, or lower). This defect indicates an abnormality in the temporal and parietal parts of the brain, which can cause brain stroke, hemorrhage, tumor, or plus collection.
- Inferior/superior defect field—A VF defect occurs in half of the VF’s upper or lower half. This defect can signal the possibility of retinal detachment or malignancy in the eye.
- Central Scotoma—A defect pattern that appears as large or small spots in the VF’s center, either right or left. This vision impairment is connected to a greater risk of central macula problems.
- Tunnel vision—A VF defect associated with glaucoma, a disease that manifests as peripheral VF loss in the early stage, constricting the field and ending up with tunnel vision before total blindness occurs.
- Normal VF—Included in the study as a baseline condition.

Until recently, studies have proposed various methods to classify specific eye diseases from various kinds of eye images, such as glaucoma [2,3,4] or diabetic retinopathy [5,6]. Thus, having a consistent and accurate framework for automatically classifying multiple patterns from eye imaging has become crucial for detecting patterns that may become indicators of several optic pathway diseases. Nowadays, other than traditional machine learning, researchers have been exploring deep learning to classify multiple diseases or defects [7,8,9]. One of them includes the work by Abu et al. (2021), which proposed a custom 10-layer CNN to classify six visual field defect patterns. Their experimental work produced high accuracy of 96% compared to other machine learning methods such as SVM and Classic Neural Network [7].

Inspired by [7], instead of designing a custom layer of CNN for visual field defect classification, the current work extends their work by focusing on the existing pre-trained CNN models (VGG-Net [10], MobileNet [11,12], ResNet [13], and DenseNet [14]) to conduct the same task. This is an approach of reusing previously developed models to solve a similar problem to speed up computation in the deep learning framework [15,16,17]. These pre-trained models were chosen because they have produced high-performance classification on eye image datasets in many previous studies [5,18,19,20]. Furthermore, CNN and pre-trained models also showed better performance in several medical image datasets, but each medical image only compared the hyperparameter in one pre-trained model [21,22,23]. Hence, more analysis of the pre-trained model will be conducted in this work. In addition, Bayesian optimization was later used to perform hyperparameter tuning and fine-tuning against all models. Hyperparameter tuning [6,24] is a search process for the ideal parameters that define the model architecture, such as epoch, batch size, optimizer, etc. As for the fine-tuning process [25,26], it is performed to adjust the layers of the pre-trained models so that the target dataset (visual field defect images) may be tuned with the source dataset and the over-fitting issue can be solved.

In this work, model optimization was performed to adjust the pre-trained models in the deep learning framework to adapt or refine the input-output pair data [15,27] and improve the classification accuracy for multiclass visual field defects. Furthermore, we perform a comparative analysis of the performances of the different pre-trained models and the effects of Bayesian optimization in choosing a suitable hyper-parameter for the pre-trained model on the visual field defect classification task. Finally, the combination of different hyperparameters and fine-tuning layers chosen during the optimization process was analyzed. The analysis aims to investigate the effect of the combination of different hyperparameters and fine-tuning layers in the pre-trained models on the classification performance of the visual field defect dataset. In this regard, the main focus of our work is to answer the following research questions:

1. What is the performance of transfer learning models in visual field defect classification?
2. What is the performance of transfer learning after applying Bayesian optimization?
3. How does a combination of different hyperparameter tuning and fine-tuning layers by Bayesian optimization affect the performance of the transfer learning models in visual field defect classification?
4. How does the fine-tuning of network layers affect the performance of the transfer learning models in visual field defect classification?

This paper is organized as follows: Section 2 presents a review of previous related works. Section 3 explains the main features of the visual field image datasets. Section 4 discusses the experimental framework for conducting the performance analysis of visual field defect classification. Section 5 reports the analysis of pre-trained models in visual field defect classification and the performance of automated hyperparameter tuning and automated fine-tuning layers on the pre-trained models using Bayesian optimization. Lastly, Section 6 presents the conclusion and potential for future research.

## 2. Related Works

This section surveys the latest literature on VF defect classification using the deep learning method. The latest assessment on automated hyperparameters and fine-tuning layers of the transfer learning method using Bayesian optimization is subsequently addressed. Finally, this section also summarizes existing knowledge on machine learning and deep learning in the classification of VF defects. In addition, it describes the image classification hyperparameters and fine-tuning process.

Previous research on VF defects has focused on detecting glaucoma [2] and the progression of glaucoma [4]. Kucur et al. [2] implemented a custom CNN with ten layers of feature learning to identify glaucoma in VF defects. They used two types of VF image datasets: Humphrey Field Analyzer 24-2 and OCTOPUS 101 G1, collected at Rotterdam Eye Hospital from 201 patients. The OCTOPUS 101 G1 dataset had an accuracy of 84.5%, whereas the Humphrey Field Analyzer 24-2 dataset had 98.5% [2]. Park et al. [4] utilized Humphrey Field Analyzer 24-2 data from Pusan National University Hospital’s glaucoma clinic (South Korea). A training dataset of 1408 images and testing of 281 images were utilized in their dataset. The deep learning method used in their work is called Recurrent Neural Network (RNN) [28]. The pattern of glaucoma change over time was also identified using RNN. The proposed method utilized RNN to detect glaucoma progression from 2005 until 2018, and the accuracy obtained was 88% [4].

Other studies on hyperparameters are limited to VF defects; however, some works have been done on retinal image datasets and other medical images. For example, Shankar et al. [6] developed a new automated Hyperparameter Tuning Inception-v4 (HPTI-v4) model for Diabetic Retinopathy (DR) detection and classification from color fundus images [6]. The MESSIDOR DR datasets [29] had undergone three processes to obtain high accuracy in their work. First, the datasets were pre-processed using contrast enhancement through CLAHE; and segmentation using a histogram. Subsequently, the features from the pre-processing process were extracted using their proposed work HITP-v4, and the output was classified using Multi-Layer Perceptron (MLP) with ten-fold cross-validation. For the best result, Bayesian optimization chose epoch: 500, learning rate: 0.001, and momentum: 0.9 during automated hyperparameter tuning. Their results were also compared with other works and showed the highest accuracy of 99.49% [6]. In the recent study on COVID-19 chest X-ray image data, M. Loey et al. (2022) developed a Bayesian optimization-based convolutional neural network to recognize COVID-19 on 10,848 chest X-ray images (3616 COVID-19, 3616 normal cases, and 3616 Pneumonia). They had built a new classifier for using CNN layers, and the layers hyperparameter will be tuned using Bayesian Optimization. They had compared their work with others and had obtained 96% accuracy [30].

A. Monshi et al. [31] developed CovidXrayNet based on the EfficientNetB0 pre-trained model to optimize data augmentation and CNN hyperparameters. Two datasets were used to evaluate CovidXrayNet: COVIDCXR (960 X-ray dataset) and Covidx (15,496 X-ray dataset). Both datasets contain three disease classes: COVID-19, pneumonia, and normal. This work optimized the data augmentation parameters based on image resize, rotate, zoom, warp, lighting, and flip. Next, the best data augmentation was tested using the transfer learning method to find the CNN hyperparameters such as epoch, batch size, and loss function for CovidCXR and Covidx. The CovidXrayNet method achieved a high accuracy of 95.82% with 30 epochs, 32 batch sizes, and cross-entropy label smoothing [31]. Another work that optimizes hyperparameters in transfer learning has been done by Loey & Mirjalili [32]. They used transfer learning to detect COVID-19 from cough sounds, and the dataset contained 1457 (755 COVID-19 and 702 healthy). ResNet-18 showed the highest accuracy of 95.33% of the chosen models, with SGD as the optimizer [32].

Besides hyperparameter tuning, the fine-tuning process is also essential in transfer learning to find the best pre-trained layer for a particular dataset. Several works have successfully developed an automated fine-tuning method to achieve the best results. Y. Wang et al. [25] improved the fine-tuning method that Y. Guo et al. [33] developed. SpotTune is a method developed by Y. Guo et al. [33] that tunes transfer learning at a specific point in the layers. Subsequently, Y. Wang et al. [25] enhanced SpotTune by tuning the transfer learning layer based on several parts of the layer and renamed the method as MultiTune. Both methods follow the adaptive fine-tuning process by using a policy network to decide if the image should be routed through fine-tuning or by using the layers of a pre-trained model. Y. Wang et al.’s [25] work, Spottune, and Multitune were compared using two types of datasets: Aircraft [34] and CIFAR100 [35]. They concluded that their method is the best by obtaining 59.59% for Aircraft, which is 4% higher than the Spottune method, and 79.31% for CIFAR100, which is 1% higher than the Spottune method. Since ResNets act like ensembles of shallow classifiers and are robust to residual block swapping [27], both approaches utilized the ResNet pre-trained model for fine-tuning.

Most studies on VF datasets using Humphrey VF image have only focused on detecting glaucoma and the progression of glaucoma patterns against time by using a custom model of deep learning. Fewer studies utilized transfer learning on VF datasets to detect multiple defect patterns from the Humphrey VF image. Therefore, pre-trained models were optimized to conduct VF image classification. Due to the lack of studies on transfer learning models for VF datasets, the potential of optimizing pre-trained models to improve VF classification performance has yet to be explored. Since the re-use of pre-trained models for solving a similar problem can speed up the computational process, further experimental studies and analysis on the optimization of transfer learning are necessary to analyze and investigate the effect of the combination of different hyperparameters and fine-tuning layers in VF defect classification. Therefore, four types of pre-trained models have been proposed based on their performance in previous work, and Bayesian optimization was used to optimize the hyperparameter in the model.

## 3. Dataset Characteristics

This work combined three datasets collected from different public dataset sources. The first dataset is the Humphrey 10-2 Swedish Interactive Threshold Algorithm (SITA) standard VF [36,37], which contains Humphrey 10-2 VF images collected from 200 patients. The second dataset is from Humphrey 24-2 in Rotterdam Eye Hospital, which consists of Humphrey 24-2 VF images from 139 patients [38,39]. In addition, the RT_dataset is from Kucur et al. [2,40], which consists of Humphrey 24-2 VF images of 161 patients. This dataset was also collected from the Rotterdam Eye Hospital. These datasets have extracted images of 1200 VF defects with six defect patterns. Table 1 shows all of the datasets collected and used to create a distribution of VF defects.

**Figure 2:**
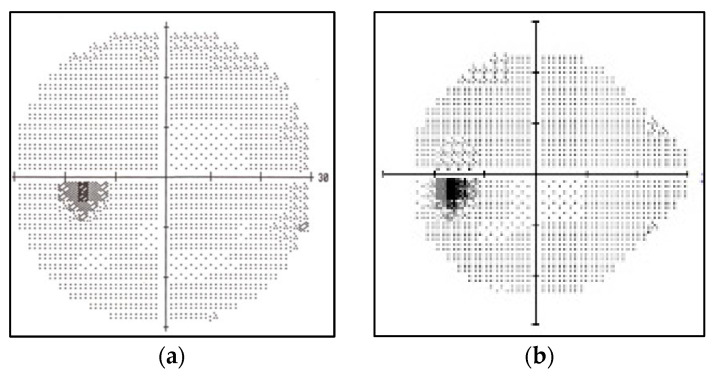
Normal VF Images: (**a**) HVF 10-2; (**b**) HVF 24-2.

There are several types of Humphrey VF testing protocols depending on the type of machine used by ophthalmologists to plot VF. The VF images utilized in this study come from two distinct testing procedures, which produce VF images with a 10-2 test grid and a 24-2 test grid, respectively. The difference between these types of VF datasets is the evaluated point plot by the machine. The 10-2 testing procedure evaluates 68 points in the central 10 degrees, and the pattern shape is similar to a circle. In contrast, the 24-2 testing procedure uses a 54-point test to assess 24 degrees temporally and 30 degrees nasally. The shape is pointy—whether on the right side or left side of the eye, depending on the eye position being tested [41]. Figure 2 depicts the VF images of normal Humphrey VF (HVF) 10-2 and normal Humphrey VF (HVF) 24-2.

Compared to other frequently used eye datasets for optical disease detection, such as fundus images and OCT images, Humphrey VFs are used to detect the optic pathway that causes visual loss. In contrast, fundus images and OCT images are used to detect the damage happening in the eye layers [41]. Therefore, these datasets were proposed to detect VF loss that cannot be detected using fundus or OCT images. In addition, various patterns of VF defects can be mapped using Humphrey VF; however, in this work, we performed classification and analysis based on the five types of defect patterns, as shown in Table 2.

The traditional examination method for identifying the defects listed in Table 2 requires experience, skills, and more time to produce consistent results when dealing with complex cases. For example, the defect patterns for central scotoma and tunnel vision show almost similar defect characteristics, as well as the defect patterns between hemianopia, quadrantanopia, and superior. Therefore, many researchers have recently explored and proposed an effective mechanism using deep learning to classify VF defects to aid physicians in producing fast, consistent, and accurate diagnostic results. However, due to the lack of datasets from VF, optimization of the transfer learning models is the most effective and efficient technique to increase the performance of VF defect classification. Therefore, several transfer learning models have been built. Thus, experimental studies and analyses are needed to compare these models and obtain the ideal hyperparameters and fine-tuned setting for the VF defect classification task.

## 4. Framework

**Figure 3:**
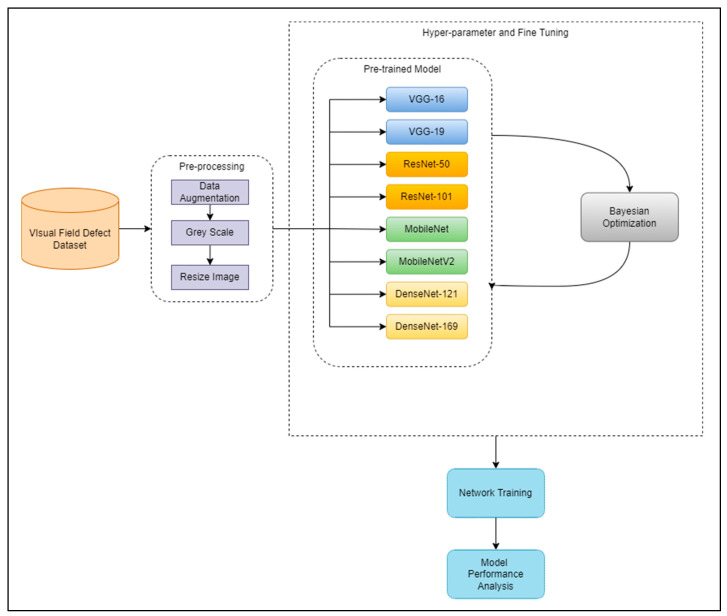
The framework of a comprehensive analysis of automated hyperparameters and automated fine-tuning for pre-trained models.

This section explains the proposed system’s framework to optimize the transfer learning models for VF defect classification and analyze their performance. First, the VF datasets must undergo pre-processing before feeding them to the pre-trained models for classification. Next, the classification of VF defects was conducted in two phases. The first phase is VF classification using 8 different pre-trained models (VGG-16 [10], VGG-19 [10], MobileNet [11], MobileNetV2 [12], ResNet-50 [13], ResNet101 [13], DenseNet-121 [14], and DenseNet-169 [14]) without performing Bayesian optimization. In the second phase, hyperparameter tuning and fine-tuning were performed against all pre-trained models using the Bayesian optimization method, and VF classification was performed, analyzed, and evaluated. The framework of the proposed work is shown in Figure 3.

### 4.1. Pre-Processing

The pre-processing method was performed on VF images using KERAS pre-processing and OpenCV as the pre-processing tools to convert the image representation. VF datasets in RGB format were converted into greyscale using OpenCV to collect information from the source image to improve image quality as much as possible [42,43].

The VF datasets are divided into two parts: right eyes and left eyes. Thus, the datasets were split and labeled as “right” and “left” to increase the number of datasets and avoid exchange during the augmentation process. The data augmentation process will generate changed copies of images in the datasets while retaining the predictive characteristics of the images. This process aims to increase the training image for VF defect classification. During data augmentation, the VF images are randomly rotated (rotations of 30, 45, and 135), cropped, and set to different brightness levels [44,45].

Finally, the images were resized to 224 × 224 and 256 × 256 using the OpenCV package. Since the datasets come from different sources, the dataset sizes also differ. A medium-sized image is most suitable for transfer learning when dealing with layer feature learning. Evidently, the 224 × 224 and 256 × 256 images demonstrated high accuracy in many previous works [26,46]. The image size is important because if the image dimensions are too small, it will affect the loss and accuracy of the model. In contrast, if the dimensions are too large, the small feature is consequently difficult to learn during the training process because the image’s resolution becomes blurred.

### 4.2. Pre-Trained Models

Several efficient pre-trained CNNs have been developed for image processing problems. However, the pre-trained models must be trained and analyzed in the input layer to transfer information from the source dataset to the target dataset. In this paper, four pre-trained models with different numbers of depths were analyzed.

**Figure 4:**
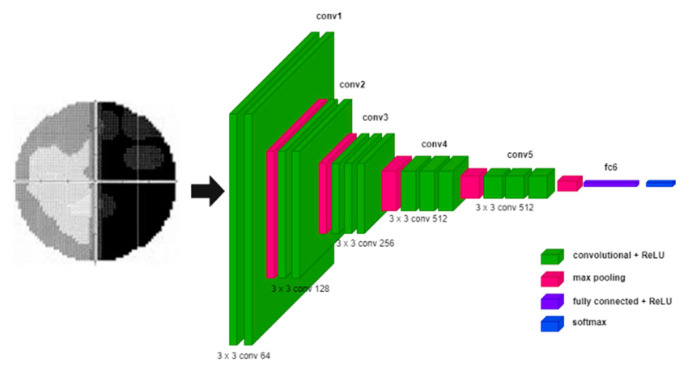
VGG-Net Model.

VGG-Net [10] is built to evaluate the increase in network depth with a small convolutional filter (3 × 3). The depths of 16 and 19 layers of VGG-Net show a significant improvement over the previous configuration. Therefore, both depths were integrated into a model known as VGG-16 and VGG-19. In VGG-Net, the input image is passed through a stack of convolutional layers [7,47] and then connected to the Max-pooling layers [2,7]. The max-pooling layers are executed over a 2 × 2 pixel window, with a stride of 2. After that, a stack of convolutional layers is followed by fully connected (FC) layers. The last layer is the Softmax layer [2,7] since this work entails multiclass image classification. The depth of VGG-Net can be changed by increasing the number of convolutional layers in conv3, conv4, and conv5. The architecture of VGG-Net is illustrated in Figure 4.

**Figure 5:**
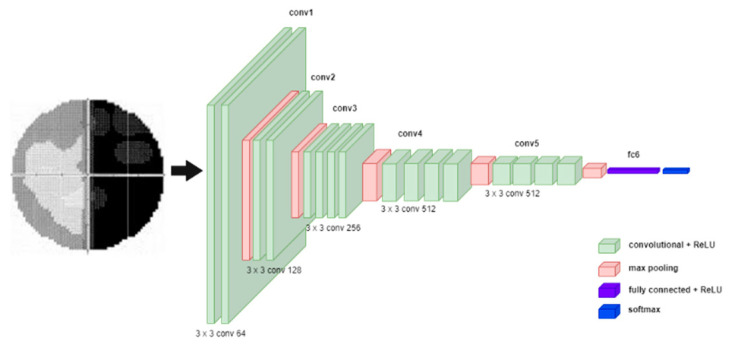
ResNet Model.

Residual Network (ResNet) [31] is structured to solve the problems associated with deep learning. The ResNet model intelligently attempts to handle several low-level, mid-level, and high-level features. The individual networks are trained to retrieve minor fragments of knowledge. The term “residual” can be understood as throwing away the functionality acquired in the previous layer. ResNet, for example, ResNet-50, ResNet-101, and ResNet-152 can be implemented in a limited layer depth, and the depth of the layer can be changed by adding or removing the convolutional layer in conv4. The number after ResNet represents the convolutional layers used in the model [31]. The convolutional layers in ResNet are concatenated into groups of convolutional to obtain multiscale maximal features from the input images. Figure 5 shows an example of the ResNet Model.

**Figure 6:**
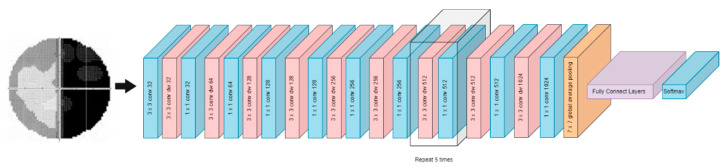
MobileNet Model.

**Figure 7:**
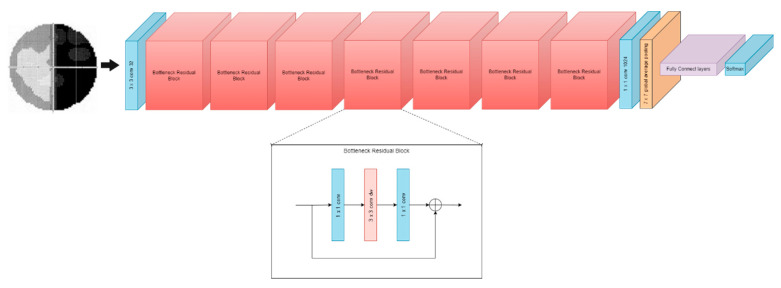
MobileNetV2 Model.

MobileNet [11] are lightweight CNN models designed for small devices like mobiles and Raspberry Pi. The structure of the MobileNet network is based on depth-separated convolutions (Conv Dw) to reduce the model parameters and shorten the training time of CNN [11]. Another version of MobileNet called MobileNetV2 [12] is based on an inverted residual structure where the linkage between the sparse bottlenecks is designed to reduce the parameters of the previous mobile architecture and make it more lightweight. In addition, the connection between the feature learning layers and fully connected layers are linked by the Global Average Pooling [47] layer in both MobileNet models, which significantly reduces the forward error estimation failure rate while reducing the model size. Figure 6 and Figure 7 show the architecture of MobileNet.

For the DenseNet [14] model, each layer is connected to every other layer in a feed-forward fashion. The feature maps of all previous layers are used as inputs for each layer, and the feature maps of the original layer are used as inputs in all subsequent layers. As a result, DenseNets [14] have several compelling advantages:

- Alleviate the vanishing gradient problem.
- Strengthen feature propagation.
- Encourage feature reuse.
- Substantially reduce the number of parameters.

**Figure 8:**
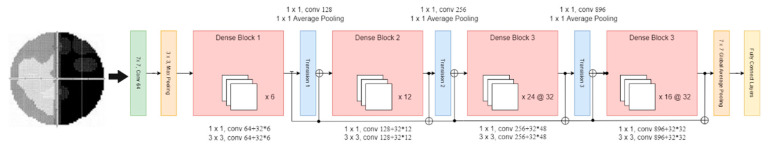
DenseNet Model.

The layers of DenseNet work similarly to ResNet, but the convolutional layer can be removed and added at Dense Block 3 and 4. Figure 8 shows the architecture of the DenseNet model. In this work, the pre-trained model’s fully connected layers are made up of flattened layers [7], dense layers [7], and dropout layers [7,48].

### 4.3. Bayesian Optimization

The Bayesian optimization technique optimizes objective functions (hyperparameters in the model architecture) that usually take only minutes or hours to evaluate compared to manual tuning [49]. This method is excellent for optimizing continuous domains with fewer than 20 dimensions, and it tolerates stochastic noise in the function evaluations [49]. Bayesian optimization is a fast method for the global optimization of unknown objective functions. Formally, it is solved using Equation (1) [6]. In this case, the maximum performance of the model was generated against a set of hyperparameters.

(1) $$x^{*}=\arg\;\underset{x\in X}{\max}\;f\left(x\right),$$

The two important processes in Bayesian optimization are the surrogate model and the acquisition function. A surrogate model is represented using the Gaussian process to fit the observed data points [50]. A Gaussian process is a useful tool for defining assumptions for smoothly changing functions in space. The Gaussian distribution’s characteristics enable us to calculate the predicted means and variances in closed form. It is defined by a mean function, *x*, and a covariance function, *k*(*x*, *x*′). The function expressed in Equation (2) [6] is a sample of a Gaussian process:

(2) $$f\left(x\right)∼GP\;\left(\mu \left(x\right),k\left(x,x^{\prime }\right)\right),$$

Bayesian optimization uses a technique similar to sequential experimental design and decision theories to predict utility. The task of the utility function is to estimate how much information a given observation can provide [51]. In Bayesian optimization, these utility functions are referred to as acquisition functions. The acquisition function helps achieve the optimum underlying function by exploring and exploiting regions where the uncertainty of the function is significant, and the predicted function values can be increased. The acquisition function can also be successfully optimized to retrieve the next point for assessment [6,52]. The acquisition function used is an expected improvement since it is assumed that this function improves the current default parameter in Bayesian to the best estimation. The expected improvement is expressed in Equation (3):

(3) $$EI\left(x\right)\equiv E\left[f\left(x\right)-f\left(x_{t}^{+}\right)\right]$$

where $x_{t}^{+}$ is the best point to observe before the next point.

**Figure 9:**
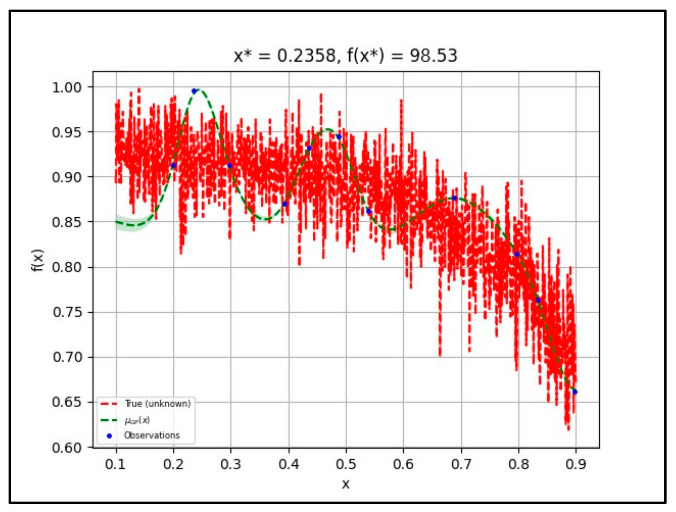
Bayesian optimization tuning dropout rate for VGG-Net.

In this case, the initial sample was well-distributed over the domain, and the surrogate function has a bias towards the part of the domain where the optimum is located. The dropout hyperparameter in transfer learning was evaluated during the optimization. Based on the default hyperparameter setting, an overabundance of samples around the known optimum point should be expected. The x function represents the input parameter (hyperparameter) and *f*(*x*) is the model (pre-trained models) used. The process of *x* and *f*(*x*) is visualized as True (unknown) in the graph, and the True (unknown) graph shows that the accuracy decreases as the dropout rate increases. The µGP (*x*) shows the acquisition function that estimates the optimum dropout rates (*x*) after every surrogate model (Gaussian Process) is fitted with the transfer learning model (*f*(*x*)). There were 11 observations; hence, the probability of obtaining the best accuracy is higher. The observations were selected by the local maximum depending on the acquisition function. The estimation of the µGP (*x*) graph follows the pattern of True (unknown). Figure 9 shows the effect of changes in the dropout hyperparameter on the model’s accuracy.

In the pre-trained learning models, several hyperparameters can be tuned for optimization. Therefore, in this work, 12 optimal hyperparameters, including the fine-tuning, were selected to be optimized by Bayesian during the training and validation process. The hyperparameters for the automated optimization include learning rate [53], filter size [7], feature maps [7], pooling [7], activation function [54], batch size [53,54], epoch [53,54], optimizer [55], and dropout rate [7,48]. The learning rate [49] is used to tune the convergence of the model’s accuracy as its loss approaches the minimum value with decreasing tendency. However, it also depends on the optimizer used. Feature maps and pooling are essential for tuning the weight of the neural network when it comes to feature learning. The hyperparameter tuning and fine tuning process will define the total parameters used by the models.

The activation function [54] suppresses irrelevant data points in the datasets. Rectified Linear Unit (ReLU) [54] and sigmoid activation [54] functions are frequently used for image classification and show good performance in previous works [54] since the image data consists of a positive number either in grayscale or RGB format. Both methods have their advantages when it comes to deep learning. In terms of the computational process, ReLU has more advantages because the computational process is simple and requires only a forward and backward pass, while sigmoid requires an exponential computation [54]. Thus, for the vanishing gradient in the graph, the sigmoid has more advantages because it is derivatively very close to zero and easier to saturate than ReLU, which saturates only when the input is less than zero [54].

The batch size determines the number of datasets that the learning algorithm goes through before changing the internal model parameters. In contrast, the epoch determines the learning algorithm’s processing time in the entire training dataset [49]. Therefore, the batch size depends on the number of datasets used in the models; it can produce a good result if the datasets are evenly distributed among the group. In a previous work [32], it was shown that the higher the epoch, the higher the accuracy—but taking into account the types and size of the dataset [53]. Therefore, the epoch and batch size were set as default parameters in this work. Bayesian optimization will choose the best epoch and batch size suitable for VF defect classification.

Besides accuracy, the loss is also an essential factor in image classification during the training phase in transfer learning. For this reason, an optimizer is used to modify the attributes of the neural network, such as weights and learning rate, to minimize losses inside the deep learning layers during training. ADAM, SGD, Adadelta, and RMSprop were utilised in this study to alter the weight and acceleration time inside the model layers of each pre-trained model. This optimizer focuses on the inner optimize in deep learning, which handles weight and bias throughout the fitting process. While Bayesian Optimization handles the outer optimization in deep learning by selecting the optimal hyperparameters for the layer feature maps, filter size, activation function, pool size, dropout, and fine tuning, Bayesian Optimization is responsible for the outer optimization. Then, epoch and batch size are optimized by Bayesian Optimization so that the highest level of accuracy may be achieved, while weight and bias are optimized by ADAM, SGD, Adadelta, or RMSprop. Thus, it explains that each optimizer serves a distinct purpose in various aspects of deep learning. A different optimizer gives a different performance depending on the model’s architecture used to classify the image. Optimizer is part of hyperparameter that will be optimized by Bayesian optimization in deep learning. Due to the different parameters and weights produced by different pre-trained models, a dropout rate is used to avoid over-fitting in the model by removing nodes in the neural network [48].

On the other hand, Bayesian optimization is performed to select the optimal combination of hyperparameters to evaluate the overall deep learning framework. The combination of hyperparameters in this work consists of 12 hyperparameters, which were combined to investigate and analyze the automated hyperparameters and the fine-tuning layers that include freezing the upper or lower network layer. The automated hyperparameters and automated fine-tuning will go through 11 evaluation processes to determine a set of optimal hyperparameters and layers that will produce high accuracy for VF defect classification. To evaluate the hyperparameter and fine tuning the pre-trained layers using Bayesian optimization, a default value for the hyperparameter and fine-tuning layers must be set to guide Bayesian optimization in selecting the optimal hyperparameter in the group area. Setting the default parameter is critical to ensure that the Bayesian optimization process achieves the correct group objective.

### 4.4. Model Evaluation

The hyperparameter in the pre-trained model optimized by Bayesian optimization is stored in the model; and then tested to obtain the accuracy, precision, recall, and *F*1 *score* using Equations (4)–(7). Equations (4)–(7) explain these metrics for generic class *k*, where *TP* refers to True Positives classifications, FN denotes False Negatives classifications, *TN* presents True Negatives classifications, and *FP* denotes False Positives classifications [31]. The value of *K* represents the number of classes.

(4) $$Accuracy=\frac{\sum_{k=1}^{K}\frac{TN_{k}+TP_{k}}{TN_{k}+TP_{k}+FN_{k}+FP_{k}}}{K}$$

(5) $$Precision=\frac{\sum_{k=1}^{K}\frac{\;TP_{k}}{TP_{k}+FP_{k}}}{K}$$

(6) $$Recall=\frac{\sum_{k=1}^{K}\frac{TP_{k}}{TP_{k}+FN_{k}}}{K}$$

(7) $$F1\;score=2\times \frac{Precision\times Recall}{Precision^{-1}+Recall^{-1}}$$

## 5. Experimental Results and Discussion

In this section, we analyze and discuss the comparison of the classification performance of VF defects based on Bayesian optimization in the transfer learning models. The VF datasets were split in a ratio of 80:10:10 for training, validation, and testing. The analysis of VF defect classification was divided into three parts. The first part consists of validating the performance of pre-trained models (VGG-16, VGG-19, ResNet-50, ResNet-101, MobileNet, MobileNetV2, DenseNet-121, and DenseNet-169) with two different image sizes: 224 × 224 and 256 × 256. The second part includes validating the performance of transfer learning after performing automated hyperparameter tuning and automated fine-tuning against all of the pre-trained models using Bayesian optimization. Finally, the last part consists of an analysis of the transfer learning performance when certain layers are frozen while performing the automated hyperparameter tuning process. The optimum result obtained from the hyperparameters and automated fine-tuning for each pre-trained model was saved in a file format to store structured data and test the accuracy of each class of VF defect. The algorithm was written in the Python language using the KERAS (Tensorflow backend) neural network computing framework. The algorithm was executed on an Intel Core i7-10 processor with 8 GB of RAM, using an RTX 2080 as the GPU.

### 5.1. Part I: Validation Results and Analysis before Bayesian Optimization

In this part, validation data was used to evaluate the pre-trained models to observe the effects of different transfer learning architectures on the VF defect datasets. For this task, the images were resized into 224 × 224 and 256 × 256 dimensions. As the image size increases, the number of parameters of the model also increases. In Table 3, the parameters without and with VF represent the parameters extracted before and after the target dataset was transferred inside the pre-trained models, respectively, and the increasing image size affects the parameters extracted by the model, in addition to increasing the layer count.

Based on the experimental results in Table 3, the 224 × 224 image size produced a good result compared to the 256 × 256 image size for most of the pre-trained models. Ideally, a larger image size contributes to a high number of parameters for the pre-trained models and increases the classification accuracy. In certain cases, however, accuracy will decrease because the larger image size introduces too many features and leads to overfitting during training and testing data. This may also apply to the increasing size of model architecture, e.g., model layers. The work of Joseph and Balaji [52] has also demonstrated that enlarging the image alone will not improve performance. Therefore, the model architecture must be highlighted to capture all features from the bottom to the top layers.

**Figure 10:**
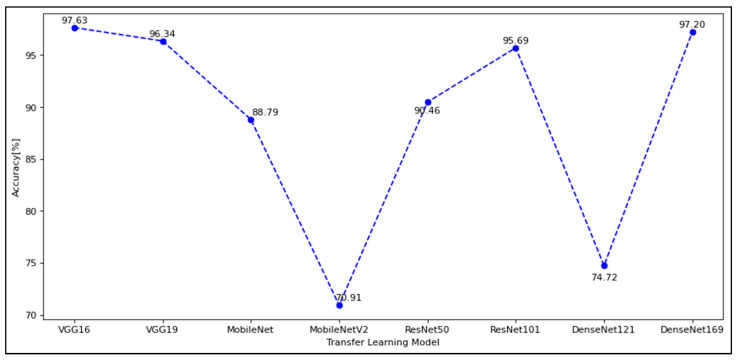
Validation of pre-trained model accuracy on a 224-image size.

From Figure 10, it can be concluded that for VF defect classification, when the number of parameters optimized by transfer learning is less than ten thousand, the highest accuracy obtained is 88% for the image size of 224 × 224. This is because underfitting occurs when the method cannot achieve higher accuracy due to insufficient parameters to process the data. In contrast, if the number of parameters for the VF datasets is too high, overfitting may occur and cause the accuracy plot to become unstable with the lowest accuracy to be selected. However, the problem can still be solved through k-fold cross-validation or by choosing an optimal hyperparameter for the models.

In this work, models with parameters above 12,000,000 achieved over 90% accuracy. With 32 batch sizes, 50 epochs, and ADAM as optimizers of pre-trained models, they can achieve high accuracy with the VGG-16 model. Since the parameters of the pre-trained models can be optimized to fit the datasets, Bayesian optimization was, therefore, used to investigate the combination of different hyperparameters and fine-tuning of the pre-trained models for the VF defect classification task.

### 5.2. Part II: Validation Results and Analysis after Bayesian Optimization

In this part, investigation and analysis were conducted to observe the effects of the combination of different hyperparameters and fine-tuning layers by a Bayesian optimizer. VF defect classification was validated with 10% of the validation of datasets. Five-fold cross-validation was applied during the optimization process to avoid validation accuracy that is higher than training accuracy (over-fitting). The best validation accuracy optimized by Bayesian optimizer was used for the testing process based on the overall framework in Figure 1 and was applied to all pre-trained models, VGG-Net, MobileNet, ResNet, and DenseNet. The hyperparameters and fine-tuning were set in a range and listed as a reference for Bayesian optimization. In this work, the hyperparameters are as follows: Convolutional layer feature maps that range from 32–64; convolutional layer filter sizes that range from 1–3; convolutional layer activation functions that include ADAM, SGD, Adadelta, and RMSprop; learning rate that ranges from 0.00001–0.1; batch size that ranges from 1–32; epoch that ranges from 10–200; dropout that ranges from 0.1–0.9.

For fine-tuning, the layers were divided into two groups: upper layer and lower layer, which were chosen by dividing the pre-trained model layers into two parts to obtain the number of layers. There are four groups of fine-tuning layers: false/false (freeze all layers), true/true (unfreeze all layers), true/false (unfreeze upper layer), and false/true (unfreeze lower layer). The 11 calls were made in Bayesian optimization to evaluate the performance of the pre-trained models. The hyperparameter tuning will be done first, then the layer will be fine-tuned to optimize the models to utilize pre-trained network weights as initialization for VF to be trained on ImageNet from the same domain. Subsequently, the weight and the parameters will be optimized and the performance will be evaluated.

The experimental results in Table 4 show the performance of each pre-trained model after optimization. The first pre-trained model optimized by Bayesian is VGG-16. The performance is above 90% when the activation set is ReLU because ReLU only picks max (0, x); therefore, the gradient is either greater than zero or less than equal to zero, while the sigmoid graph approaches zero and tends to vanish the gradient. Besides, some hyperparameters choose ReLU as the activation function, but the accuracy is low due to the optimizer’s effect and that the learning rate for the optimizer causes the optimizer to set the limit for training accuracy as a result of no improvement in the testing process.

For the epoch in VGG-16, the larger the epoch size, the higher the accuracy; however, this also depends on other hyperparameters such as dropout. The accuracy decreases if the dropout is too large for the network. Meanwhile, the three hyperparameters of the feature map, filter size, and pooling size are interrelated for producing the number of parameters for the model through calculation (width of filter size × height of filter size × the number of previous filters + 1) × the number of feature maps). Each layer of the convolutional neural network will be linked to each other until the classification layer is approached. The larger the number of extracted parameters, the higher the degree of accuracy. As for the pre-trained VGG-19 model, the activation function part is the same as the VGG-16 model, which means that ReLU shows the best performance compared to sigmoid. Regarding the optimizer and the learning rate, it was observed that the high learning rate chosen for ADAM did not lead to any improvement in accuracy above 20.69%.

The MobileNet model has the lowest accuracy compared to other models, even after automatic tuning of hyperparameters and fine-tuning. Although the small number of parameters has caused underfitting for this model, MobileNet is still lightweight compared to others. After Bayesian optimization, the results showed that MobileNet’s performance increased by 5% for MobileNet and 23% for MobileNetV2. Thus, since the magnitude of the gradients may vary depending on the weight of the network, the RMSprop optimizer has shown high accuracy for the MobileNet model with an appropriate learning rate. This is because RMSprop addresses this problem by maintaining a moving average of the quadratic gradient and adjusting the updates to the weights by this magnitude [53].

In ResNet, both ResNet50 and ResNet101 showed stably high accuracy for most of the hyperparameter tuning and fine-tuning processes. This is because ResNet adds shortcuts between layers to avoid distortions that occur in deeper and more complex networks [54]. From Table 4, it can be seen that ResNet with low accuracy below 25% has a small number of kernel sizes and sigmoid as the activation function. Since ResNet dominates the low-level to high-level features, the decreasing kernel size of a convolutional layer can lead to the reduction of parameters; thus, the feature extraction is reduced again and disturbs the performance of the model. Besides, the Sigmoids have a tendency to make the gradient disappear. However, this can be avoided by choosing an appropriate optimizer and learning rate, which helps maximize the efficiency of the output.

The DenseNet model also performed well for both DenseNet 121 and Dense-Net 169. As explained in Section 4, the architecture of ResNet and DenseNet is a combination of convolutional layers and pooling layers, but the way they combine the layers is different. ResNet uses summation to combine all the preceding feature maps, while DenseNet concatenates them all [55]. Interestingly, DenseNet has shown better accuracy after hyperparameters and fine-tuning because it has a higher capacity with multilayer features during concatenation. However, the problem is that the more layers are used, the more training time is required because the number of parameters is larger compared to ResNet. As for ResNet, summing layers causes the performance to become lighter and stabilize at high performance; however, this still depends on the dataset used. Hyperparameters and fine-tuning help increase the performance of the DenseNet layer, especially the optimizer and the learning rate. As for DenseNet, the Adadelta optimizer shows a high performance of over 98% due to its robustness to a large network architecture [56].

The combination of different hyperparameters and fine-tuning layers has a great impact on the transfer learning models, especially on the activation function and optimizer with the learning rate. Based on our experiment, the ReLU activation function gave the best overall performance. However, as for the optimizer and learning rate, it depends on the model structure used to classify VF defects. ADAM performed well in the VGG-Net model because it uses adaptive learning rate performance to find the individual rates for each parameter, and the VGG-Net structure consists only of convolutional layers and pooling layers for ADAM to evaluate each parameter in each layer. On the contrary, optimizers such as RMSprop and Adadelta performed extremely well on complex structures such as ResNet and DenseNet. On the other hand, SGD produces a good performance rate only if a suitable learning rate is set. In terms of convolutional layer feature maps, filter size and pooling, it can be concluded that the larger the hyperparameters, the higher the accuracy. However, the number of filter sizes and pooling sizes must not be too large; otherwise, they will exceed the size of the feature maps and cannot be processed.

As for the batch size, the accuracy of VF detection increases when the batch size is larger for ADAM and SGD optimizers; however, for RMSprop and Adadelta, the batch size should be small. The relationship between the batch size and the optimizer was found in the curve plot between the number of epochs and accuracy. As for ADAM and SGD, the higher the batch size, the less stepper the graph is drawn. Meanwhile, for RMSprop and Adadelta, the higher the batch size, the more stepper the graph is plotted.

This process is called an asymptotic behavior and this problem can also be applied to the learning rate in the optimizer; however, in the learning rate, the graph becomes stepper when the batch size is larger, showing that the model has difficulty in achieving high accuracy [57]. In terms of the epoch, it can be concluded that the larger the epoch, the higher the accuracy—up to a certain limit before overfitting occurs.

The automated hyperparameter tuning and automated fine-tuning using Bayesian optimization in the four pre-trained models (VGG-Net, ResNet, MobileNet, and DenseNet) were carried out to investigate and analyze the effects of the combination of different hyperparameters and fine-tuning layers on each model. Each pre-trained model was divided into two different numbers of layers: VGG-16, VGG-19, ResNet50, ResNet101, MobileNet, MobileNetV2, DenseNet-121, and DenseNet-169. Based on the experiment setup in Table 4, the automated hyperparameter tuning and automated fine-tuning using Bayesian optimization were able to achieve high accuracy above 90%. However, the specific impact of each hyperparameter tuning and the fine-tuning process is unknown.

During validation of automated hyperparameter tuning and automated fine-tuning using Bayesian optimization, VGG-16, ResNet-50, MobileNetV2, and DenseNet-121 showed the highest accuracy for each pre-trained model. However, based on the experiment results in Table 4, since hyperparameters and fine-tuning were experimented together, it is unclear which fine-tuning has the greatest impact on the models. Therefore, a separate experiment with four fine-tuning processes (freezing all layers, freezing upper layers, freezing lower layers, and thawing all layers) was conducted using the Bayesian optimizer. In this experiment, the performance of different fine-tuning and automatic hyperparameter optimizations was evaluated based on the convergence plot.

**Figure 11:**
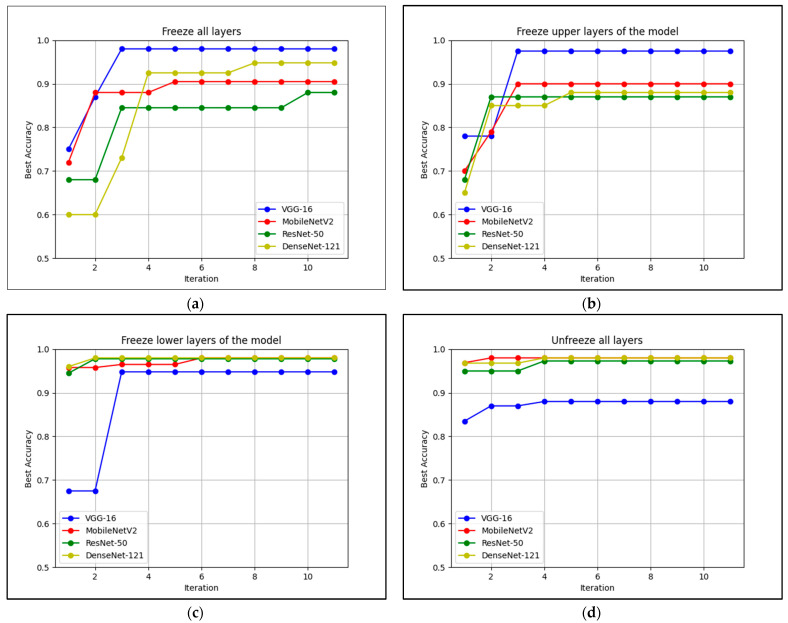
Validation of pre-trained models when some layers were frozen: (**a**) Freeze all layers (the upper and lower layers of the model are FALSE); (**b**) Freeze upper layers of the model (the upper layers are FALSE and the lower layers are TRUE); (**c**) Freeze lower layers of the model (the upper layers are TRUE and the lower layers are FALSE; (**d**) Unfreeze all layers of the model (the upper and lower layers are TRUE.

Figure 11 shows the performance of pre-trained models with four different fine-tuning layers. To perform fine-tuning, the pre-trained layers were frozen (untrainable) and not frozen (trainable). First, all layers in the pre-trained models were frozen and only VGG-16 and MobileNetV2 achieved accuracy above 90%, while ResNet-50 and DenseNet-121 achieved accuracy below 90%. VGG-16 performed best when all layers were frozen, achieving the highest accuracy and tuned only twice by Bayesian.

Second, validation was performed while the upper layers of the pre-trained models were frozen. The performance of the models did not change significantly, except that the accuracy of the performance decreased slightly compared to when all layers were frozen. Despite the slight degradation in performance, Bayesian required fewer calls to achieve the best accuracy when the upper layers were frozen instead of all layers. Third, validation was performed while the lower layers of the pre-trained model were frozen. As a result, ResNet-50, DenseNet-121, and MobileNetV2 achieved high accuracy above 90% in the first round of tuning and the best accuracy above 95% in the second round of tuning for ResNet-50 and DenseNet-121. Finally, validation was performed while all pre-trained layers were not frozen. The results showed that ResNet-50, MobileNetV2 and DenseNet-121 achieved a high accuracy of 95%, except for VGG-16, which achieved about 80%.

It can be concluded that the performance is best when the lower layer is not frozen. This is because the lower part of the feature layers is combined with the fully linked layer, and this shows that fine-tuning has a significant impact on the models based on the datasets used. Besides, it was observed that the upper parts of the feature layers have large feature maps, which leads to the need to update more weights and parameters. A sudden large feature map set for the layer could also have led to overfitting.

However, for VGG-16, by freezing all layers, the model performed well because the layers consist of only one convolutional layer and one max-pooling layer, making the extracted features and weights from the source data compatible with the target data. Table 3 shows that the parameters extracted from VGG-16 are neither too large nor too small when trained with the source data. Freezing all layers in this model is not efficient because it is time-consuming and some of the previously set weights are already suitable for the target data set. Excessive retraining of the model may lead to overfitting. In conclusion, the fine-tuning of the network layers have specific effects on the validation performance of each model, which are mainly influenced by the architecture of the model itself.

### 5.3. Part III: Classification Results and Analysis after Bayesian Optimization

Previously, these datasets were tested with a ten-layer convolutional neural network model based on a previous study by Kucur et al. [2], which achieved an accuracy of 96%. Therefore, the same datasets were used to analyze the performance of VF defects in the transfer learning framework with automated hyperparameter tuning and automated fine-tuning by Bayesian optimization. The performance of this work in a 10% test data was computed in accuracy, precision, F1 score, and recall of VF defects in distinguishing between the six classes of VF defects (central, hemianopia, normal, quadrantanopia, superior, and tunnel).

The cross-entropy loss function was used in this study because it is most commonly used for classification problems. Evidently, automated hyperparameter tuning and automated fine-tuning can fit the parameters and weights of the models to the datasets and obtain high accuracy for each pre-trained model. The performance results are shown in Table 5. In this work, after hyperparameter tuning and fine-tuning, the performance of DenseNet-121 reached similar accuracy to the work of Kucur et al., which performed classification of only two types of disease, glaucoma and nonglaucoma, from the RT dataset. With the exception of pre-trained MobileNet models, which have more parameters than MobileNetV2, most other models achieved accuracy greater than 95%.

**Figure 12:**
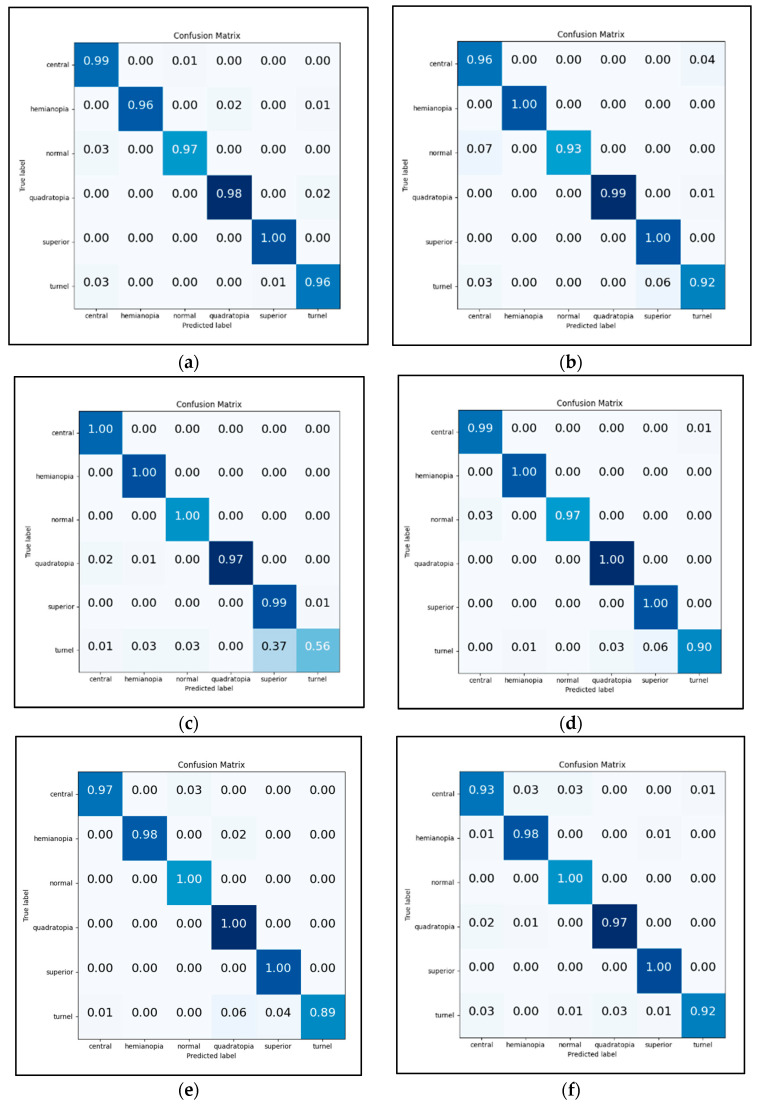

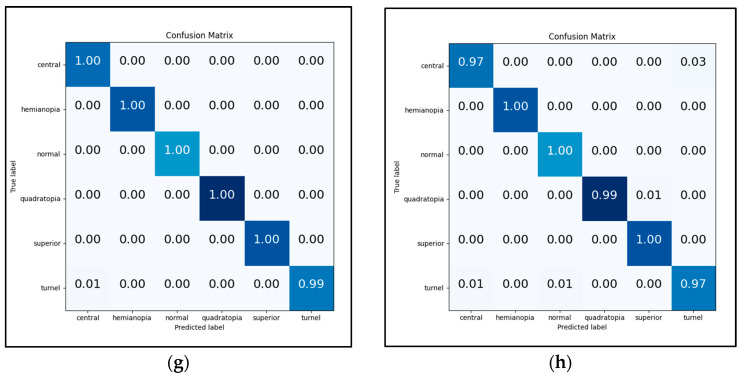
Confusion matrices for VGG-Net, MobileNet, ResNet, and DenseNet: (**a**) Confusion matrix for VGG-16; (**b**) Confusion matrix for VGG-19; (**c**) Confusion matrix for MobileNet; (**d**) Confusion matrix for MobileNetV2; (**e**) Confusion matrix forf ResNet-50; (**f**) Confusion matrix for ResNet-101; (**g**) Confusion matrix for DenseNet-121; (**h**) Confusion matrix for DenseNet-169.

For each pre-trained model, the optimal tuning of the model determined by Bayesian optimization was used. The precision of each VF class by different pre-trained models is shown in Table 5. In this work, six different types of VF defects were classified and Figure 12 shows the confusion matrices for the six groups (central, hemianopia, normal, quadrantanopia, superior, and tunnel) from the classification. The test confusion matrix is shown in Figure 12a,b for the two VGG-Net models (VGG-16 and VGG-19), with an overall accuracy of 98.28% and 97.84%, respectively.

For VGG-16, superior achieved a precision of 100%, quadrantanopia achieved 98% with 2% misclassification as tunnel vision, hemianopia achieved 96% with 3% misclassification as quadrantanopia and tunnel vision, and normal achieved 97% with 3% misclassification as central scotoma. On the other hand, VGG-19 attained a precision of 100% for superior and hemianopia, 99% for quadrantanopia with 1% misclassification as tunnel vision, 96% for normal with 4% misclassification as tunnel vision, and 92% for tunnel vision with 6% misclassification as superior and 3% misclassified as a central scotoma.

The overall accuracy for the two MobileNet models (MobileNet and MobileNetV2) is 92.45% and 97.84%, respectively. In MobileNet, central scotoma, hemianopia, and normal have a precision of 100%, quadrantanopia has a precision of 97% with 2% misclassification as central scotoma and 1% as hemianopia, superior has a precision of 99% with 1% misclassification as tunnel vision, and lastly, tunnel vision has a precision of 56% with 37% misclassification as superior, 3% as hemianopia and normal, and 1% as central scotoma. Meanwhile, MobileNetV2 has a precision of 100% for hemianopia, superiority, and quadrantanopia, 99% for central scotoma with 1% tunnel misclassification, 97% for normal with 3% central scotoma misclassification, and finally 90% for tunnel vision with 6% misclassification as superiority, 3% as quadrantanopia, and 1% as hemianopia.

The overall accuracy of the two ResNet models (ResNet-50 and ResNet-101) is 97.41% and 96.55%, respectively, as shown in Figure 12e,f. In ResNet-50, normal, quadrantanopia, and superior achieved a precision of 100%, hemianopia achieved a precision of 98% with 2% misclassification as quadrantanopia, central scotoma achieved a precision of 97% with 3% misclassification as normal, and tunnel vision has a precision of 89% with 4% misclassification as superior, 6% as quadrantanopia, and 1% as central scotoma. Meanwhile, ResNet-101 has a precision of 100% for normal and superior, 98% for hemianopia with 1% misclassification as superior and central scotoma, 97% for quadrantanopia with 2% misclassification as central scotoma, and 1% as hemianopia, 93% for central scotoma with 3% misclassification as central scotoma and 4% misclassification as normal vision, and precision of 92% for tunnel vision with 3% misclassification as quadrantanopia and 3% misclassification as a central scotoma.

In Figure 12g,h, the overall accuracy for the two DenseNet models (DenseNet-121 and DenseNet-169) is 99.57% and 98.92%, respectively. In DenseNet-121, five types of defects attained a precision of 100%: central scotoma, hemianopia, normal, quadrantanopia, and superior, except for tunnel vision, which obtained a precision of 99% with 1% misclassification as a central scotoma. As for DenseNet-169, a 100% precision was obtained for hemianopia, normal, and superior. For quadrantanopia, a 9% precision was obtained with 1% misclassification as superior. A 97% precision was achieved for central scotoma with 3% misclassification as tunnel vision, while a 99% precision was achieved for tunnel vision with 1% misclassification as central scotoma and normal. From Table 5, it can be concluded that automated hyperparameter tuning and automated fine-tuning have a significant impact on the classification performance by achieving more than 90% of precision for most pre-trained models with small misclassification for each class.

**Figure 13:**
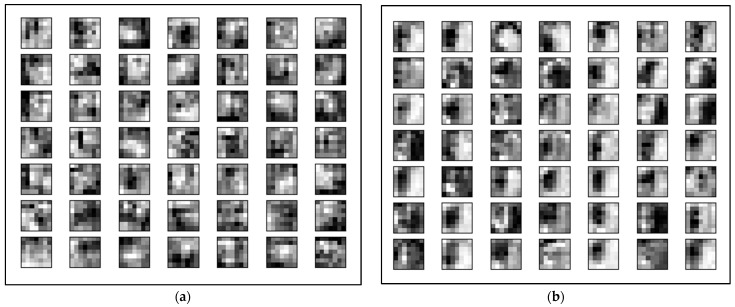
Feature learn by DenseNet-121 before and after Bayesian: (**a**) Before applying Bayesian optimization; (**b**) After applying Bayesian optimization.

Figure 13 shows the feature learned by the best pre-trained model, DenseNet-121, before and after automated optimization. Feature learning is part of the deep learning framework where a set of techniques is applied to learn a feature—a representation of raw input data before the classification. As can be seen in the figure, the different hyperparameters used in transfer learning will affect the feature learned by the layers. The image input in Figure 13a shows the features of hemianopia defects before automated hyperparameter tuning and tuning. In the figure, the features are blurred, and the structure of some images is difficult to be classified as hemianopia. On the contrary, Figure 13b shows clearer features that represent the image of hemianopia after optimization.

## 6. Conclusions and Future Works

Different transfer learning models show different performance rates in classifying VF defects. An experiment before hyperparameter tuning and fine-tuning was performed for the transfer learning models, and the VGG-16 pre-trained model showed the best performance of 97.63% compared to other pre-trained models based on 32 batch sizes, 50 epochs, and ADAM as the optimizer. Since the hyperparameters and fine-tuning layers are the important aspects of transfer learning, various combinations of different hyperparameters and fine-tuning layers were tested. The experimental results showed that the optimal choice of hyperparameters and fine-tuning could improve the performance of pre-trained models. However, when an inappropriate tuning is chosen, the classification performance decreases. To avoid this problem and reduce the time for selecting an optimal hyperparameter and fine-tuning layer, Bayesian optimization of hyperparameters can be used.

In this work, it was observed that Bayesian optimization helps improve the pre-trained ResNet, MobileNet, and DenseNet models to achieve accuracy above 90% by combining multiple optimal hyperparameters and fine-tuning based on the Bayesian optimization process. A comprehensive analysis has shown that each hyperparameter and fine-tuning has its role in processing the weights and parameters in the pre-trained models. The optimal tuning of hyperparameters in the pre-trained models helps reduce the loss during training and validation by changing the complexity of the transfer learning architecture. Based on the comparison of the combination of different hyperparameters and fine-tuning layers, the DenseNet-121 pre-trained model is the best model as it achieved a high accuracy of 98.46% during validation and 99.57% during testing. In addition, fine-tuning, i.e., freezing an appropriate network layer during validation, is also important as it helps to reduce the time required to train the target dataset. This requires extensive analysis to determine which network layers should be trained to achieve optimal results. Figure 11 shows that the right choice in freezing the layers can reduce the evaluation time and help the models achieve high accuracy of over 90%.

There are also some limitations when it comes to optimizing the hyperparameters and fine-tuning the transfer learning framework. The image size must be at least 224; otherwise, the input dimension becomes negative if the size is smaller than 224. On the other hand, if the images are resized to a larger size, they will be stretched and may become blurred. This can affect certain features of the images that are critical for medical images. In addition, the large size of the images can cause higher memory requirements on the hardware. Therefore, the development of transfer learning that can handle small images is proposed in future works. Combination of difference model can be apply but not recommended because different model have different layers function so removing layers or change layers by using automated hyperparameters optimization from the original transfer learning can also be one of the ways to deal with small images. Since some transfer learning layers are incompatible with the data set, it is preferable to remove and replace layers rather than fine-tune to minimize memory issues.

## Figures and Tables

**Table 1:** Distribution of VF Defects from Collected Datasets.

Type of VF Defect	No. of Record
Central scotoma	188
Right/Left hemianopia	205
Right/left/upper/lower quadrantanopia	150
Normal	273
Tunnel vision	207
Superior/inferior defect field	177

**Table 2:** Types of VF Defects.

Defect Type	VF Image
Central scotoma	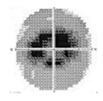 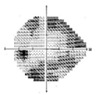 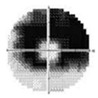
Right/left hemianopia	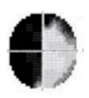 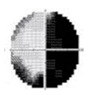 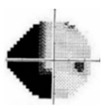
Right/left/upper/lower quadrantanopia	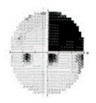 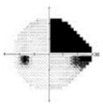 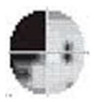
Tunnel vision	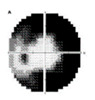 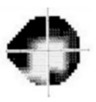 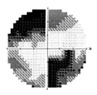
Superior/inferior defect field	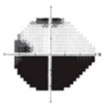 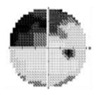 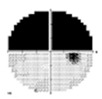

**Table 3:** Parameters of each pre-trained model.

Model	Image Size	Parameter		Validation Accuracy (%)
		without VF	with VF	
VGG-16	224	14,714,688	14,865,222	97.63
	256		14,911,302	96.55
VGG-19	224	20,024,384	20,174,918	96.34
	256		20,220,998	17.69
MobileNet	224	3,228,864	3,529,926	88.79
	256		3,622,086	94.41
MobileNetV2	224	2,257,984	2,634,310	70.91
	256		2,749,510	39.24
ResNet50	224	23,587,712	24,189,830	90.46
	256		24,374,150	86.66
ResNet101	224	42,658,176	43,260,294	95.69
	256		43,444,614	92.91
DenseNet121	224	7,037,504	7,338,566	74.72
	256		7,430,726	94.20
DenseNet169	224	12,642,880	13,132,102	97.20
	256		13,281,862	93.27

**Table 4:** Validation of automated hyperparameter tuning and automated fine-tuning of each pre-trained model.

Model	Hyperparameter									Fine-Tuned		Validation Accuracy (%)
	Feature Map	Filter Size	Activation Function	Pool Size	Optimizer	Learning Rate	Batch Size	Epoch	Dropout Rate	Upper Layer	Lower Layer	
VGG-16	64	3	ReLU	2	ADAM	0.001	32	200	0.2	FALSE	FALSE	97.72
	43	2	Sigmoid	1	RMSprop	0.0002	29	42	0.6	TRUE	TRUE	20.69
	52	2	ReLU	2	ADAM	0.0006	19	54	0.7	FALSE	FALSE	98
	48	1	Sigmoid	1	RMSprop	0.0161	27	92	0.2	TRUE	FALSE	20.69
	53	2	Sigmoid	2	ADAM	0.0081	15	103	0.8	FALSE	TRUE	17.24
	52	2	Sigmoid	2	Adadelta	0.0507	18	69	0.3	TRUE	TRUE	20.69
	39	3	Sigmoid	2	RMSprop	0.0513	11	13	0.6	TRUE	TRUE	18.1
	53	1	ReLU	1	ADAM	0.0046	9	11	0.8	FALSE	FALSE	20.69
	55	3	ReLU	1	Adadelta	0.0813	31	24	0.3	FALSE	TRUE	93.97
	34	2	Sigmoid	2	RMSprop	0.0002	15	66	0.8	TRUE	TRUE	20.69
	32	2	ReLU	1	SGD	0.0001	1	200	0.1	TRUE	FALSE	95.73
VGG-19	64	3	ReLU	2	ADAM	0.001	32	200	0.2	FALSE	FALSE	20.69
	60	2	Sigmoid	2	ADAM	0.0031	9	144	0.3	TRUE	FALSE	20.69
	60	2	Sigmoid	1	SGD	0.0082	23	169	0.6	TRUE	FALSE	20.69
	33	2	ReLU	2	ADAM	0.0004	31	166	0.4	FALSE	FALSE	97.84
	35	2	ReLU	1	SGD	0.0338	23	57	0.2	TRUE	TRUE	93.84
	46	1	Sigmoid	1	ADAM	0.0008	19	163	0.1	FALSE	FALSE	20.69
	54	2	ReLU	2	RMSprop	0.0048	30	193	0.5	FALSE	FALSE	20.69
	40	3	ReLU	2	ADAM	0.0248	6	101	0.7	TRUE	FALSE	20.69
	35	2	Sigmoid	1	ADAM	0.0002	30	73	0.2	FALSE	FALSE	20.69
	40	3	ReLU	1	RMSprop	0.0046	31	36	0.1	TRUE	TRUE	20.69
	41	1	ReLU	1	RMSprop	0.0002	13	79	0.8	TRUE	FALSE	53.28
MobileNet	64	3	ReLU	2	ADAM	0.001	32	200	0.2	FALSE	FALSE	93.58
	45	2	Sigmoid	2	SGD	0.0322	15	96	0.6	TRUE	FALSE	18.44
	60	3	Sigmoid	1	RMSprop	0.0018	18	138	0.3	FALSE	FALSE	29.26
	41	2	Sigmoid	2	Adadelta	0.0003	9	61	0.8	TRUE	TRUE	20.69
	48	2	Sigmoid	2	ADAM	0.0002	9	135	0.4	TRUE	FALSE	15.51
	57	1	ReLU	2	Adadelta	0.0055	7	106	0.2	TRUE	FALSE	94.4
	45	2	ReLU	2	Adadelta	0.001	13	154	0.4	TRUE	FALSE	93.62
	57	3	Sigmoid	1	SGD	0.0001	20	65	0.3	TRUE	FALSE	20.69
	32	2	Sigmoid	1	ADAM	0.0531	2	45	0.9	FALSE	FALSE	20.69
	57	1	ReLU	2	RMSprop	0.0005	32	21	0.4	TRUE	TRUE	95.13
	52	2	Sigmoid	1	RMSprop	0.0319	5	133	0.2	FALSE	FALSE	17.16
MobileNetV2	64	3	ReLU	2	ADAM	0.001	32	200	0.2	FALSE	FALSE	92.5
	58	1	ReLU	1	RMSprop	0.0053	14	16	0.7	FALSE	FALSE	20.82
	49	3	ReLU	1	Adadelta	0.0009	17	189	0.8	TRUE	FALSE	36.42
	44	3	ReLU	1	RMSprop	0.0003	26	43	0.4	TRUE	TRUE	86.47
	44	3	ReLU	1	SGD	0.01	14	65	0.6	TRUE	TRUE	81.77
	36	2	ReLU	2	RMSprop	0.0001	11	67	0.4	FALSE	FALSE	95.6
	64	2	ReLU	2	RMSprop	0.0134	7	119	0.7	FALSE	TRUE	20.69
	51	2	Sigmoid	2	RMSprop	0.0015	24	181	0.1	TRUE	FALSE	74.05
	51	1	Sigmoid	1	RMSprop	0.0006	6	168	0.5	TRUE	TRUE	76.38
	52	2	ReLU	2	SGD	0.0022	14	66	0.3	FALSE	FALSE	52.93
	46	3	ReLU	1	RMSprop	0.0003	21	33	0.1	FALSE	TRUE	84.01
ResNet-50	64	3	ReLU	2	ADAM	0.001	32	200	0.2	FALSE	FALSE	97.33
	63	3	ReLU	2	RMSprop	0.0601	25	101	0.4	TRUE	TRUE	47.85
	50	3	ReLU	1	RMSprop	0.0005	30	174	0.8	FALSE	TRUE	97.28
	50	1	Sigmoid	1	ADAM	0.0051	16	18	0.8	FALSE	FALSE	21.25
	54	2	Sigmoid	1	ADAM	0.0048	7	157	0.2	FALSE	TRUE	74.18
	41	1	Sigmoid	2	ADAM	0.0364	24	129	0.3	FALSE	TRUE	22.63
	45	1	ReLU	1	RMSprop	0.0189	13	12	0.3	FALSE	TRUE	85.6
	41	2	Sigmoid	2	Adadelta	0.0142	11	66	0.9	TRUE	TRUE	97.46
	50	2	ReLU	2	ADAM	0.0017	23	178	0.3	TRUE	TRUE	95.6
	46	2	ReLU	2	Adadelta	0.0049	25	13	0.8	TRUE	FALSE	75
	51	2	ReLU	2	RMSprop	0.0076	32	200	0.1	TRUE	TRUE	96.25
ResNet-101	64	3	ReLU	2	ADAM	0.001	32	200	0.2	FALSE	FALSE	96.07
	42	2	ReLU	2	RMSprop	0.061	15	110	0.6	FALSE	TRUE	75.47
	52	2	Sigmoid	1	RMSprop	0.003	32	106	0.2	FALSE	TRUE	19.01
	61	3	ReLU	2	Adadelta	0.0023	20	54	0.7	TRUE	FALSE	93.36
	45	2	Sigmoid	2	ADAM	0.0001	10	87	0.3	FALSE	FALSE	45.13
	36	2	Sigmoid	1	SGD	0.0029	19	133	0.1	FALSE	TRUE	96.67
	38	2	ReLU	1	ADAM	0.0011	12	130	0.7	TRUE	FALSE	96.29
	44	3	ReLU	2	SGD	0.0002	16	182	0.6	FALSE	TRUE	96.8
	58	2	Sigmoid	2	SGD	0.0001	18	32	0.4	FALSE	FALSE	96.77
	32	2	Sigmoid	2	Adadelta	0.1	18	66	0.9	TRUE	FALSE	93.92
	36	1	ReLU	2	ADAM	0.0016	7	113	0.1	TRUE	FALSE	96.94
DenseNet-121	64	3	ReLU	2	ADAM	0.001	32	200	0.2	FALSE	FALSE	97.96
	63	1	Sigmoid	1	ADAM	0.0329	24	61	0.1	TRUE	TRUE	71.38
	38	2	Sigmoid	2	RMSprop	0.0687	22	38	0.8	FALSE	FALSE	83.58
	44	1	ReLU	2	SGD	0.0324	9	167	0.7	FALSE	TRUE	97.89
	41	2	Sigmoid	2	ADAM	0.0003	15	67	0.6	FALSE	FALSE	76.59
	51	3	ReLU	2	Adadelta	0.0091	17	195	0.8	FALSE	FALSE	98.45
	49	1	ReLU	1	Adadelta	0.0333	11	85	0.7	FALSE	TRUE	98.06
	60	2	ReLU	1	ADAM	0.0217	18	111	0.1	TRUE	FALSE	89.44
	34	1	Sigmoid	2	SGD	0.0024	10	136	0.4	TRUE	FALSE	82.46
	47	2	Sigmoid	1	RMSprop	0.01	14	67	0.7	TRUE	FALSE	95.39
	55	2	Sigmoid	2	ADAM	0.0044	18	142	0.7	TRUE	FALSE	76.72
DenseNet-169	64	3	ReLU	2	ADAM	0.001	32	200	0.2	FALSE	FALSE	94.27
	52	2	ReLU	1	ADAM	0.0183	25	52	0.5	FALSE	FALSE	87.93
	40	2	Sigmoid	1	RMSprop	0.0108	14	69	0.8	TRUE	TRUE	96.29
	38	2	ReLU	1	Adadelta	0.0022	24	143	0.3	FALSE	FALSE	98.43
	52	2	ReLU	2	ADAM	0.0005	11	188	0.3	TRUE	TRUE	97.76
	43	3	ReLU	1	ADAM	0.0013	24	76	0.2	TRUE	TRUE	96.85
	42	1	ReLU	2	ADAM	0.0004	19	156	0.6	FALSE	TRUE	97.93
	54	2	ReLU	1	RMSprop	0.0049	4	176	0.8	FALSE	FALSE	91.98
	45	3	Sigmoid	2	RMSprop	0.0003	25	20	0.5	FALSE	FALSE	52.84
	49	1	Sigmoid	1	SGD	0.0061	2	178	0.4	FALSE	FALSE	96.9
	35	3	ReLU	1	ADAM	0.0621	19	109	0.4	TRUE	FALSE	16.72

**Table 5:** Comparison of the testing results of automated hyperparameter tuning and automated fine-tuning for the pre-trained models.

Method	Precision (%)	Recall (%)	F1 (%)	Accuracy (%)	Loss
VGG-16	97.66	97.66	97.50	98.28	0.0760
VGG-19	96.66	96.83	96.66	97.84	0.1701
MobileNet	92.00	93.83	91.50	92.45	0.3170
MobileNetV2	97.66	97.93	97.66	97.84	0.3087
ResNet-50	97.33	97.83	97.33	97.41	0.0792
ResNet-101	96.66	96.33	96.33	96.55	0.1346
DenseNet-121	99.83	99.83	99.66	99.57	0.0048
DenseNet-169	98.83	98.83	98.66	98.92	0.0774

## Data Availability

In this study, we used publicly available VF defect images, the Rotterdam ophthalmic Data Repository [http://www.rodrep.com/longitudinal-glaucomatous-vf-data---description.html (accessed on 23 September 2021)], S1-Dataset, Github dataset [https://github.com/serifeseda/early-glaucoma-identification (accessed on 29 September 2021)], and 10-2 Humphrey SITA dataset [https://datasetsearch.research.google.com/ (accessed on 23 September 2021)]. The VF defects can be made available for reasonable requests by contacting the corresponding authors.
